# Real‐World Outcomes of Immune Checkpoint Inhibitors in Melanoma, Including Acral and Mucosal Subtypes: A Retrospective Cohort Study From a Latin American Center

**DOI:** 10.1155/jskc/3904810

**Published:** 2026-09-28

**Authors:** Vaneza Avila-Rodriguez, Juan Manuel Vasquez, Luisana Molina-Pimienta, Carlos Bonilla-Gonzalez, Julian Rondon-Carvajal, Sebastian Gil-Tamayo, Camilo Rueda, Andrea Russi, Ricardo Bruges

**Affiliations:** ^1^ Hospitalization Unit, Luis Carlos Sarmiento Angulo Cancer Treatment and Research Center (CTIC), Bogotá, Colombia; ^2^ GIGA Research Group, CTIC/Universidad El Bosque, Bogotá, Colombia; ^3^ Faculty of Medicine, Pontificia Universidad Javeriana, Bogotá, Colombia, javeriana.edu.co; ^4^ Clínica Universitaria Colombia, Keralty Group, Bogotá, Colombia; ^5^ Gastrointestinal Tumors Unit, Luis Carlos Sarmiento Angulo Cancer Treatment and Research Center (CTIC), Bogotá, Colombia; ^6^ Pulmonology Program, School of Medicine, Universidad CES, Medellín, Colombia, ces.edu.co; ^7^ CardioVID Cardiovascular Institute, Medellín, Colombia; ^8^ Department of Internal Medicine, Hospital Universitario San Ignacio, Bogotá, Colombia, husi.org.co; ^9^ Hospital Universitario Mayor–Méderi, Bogotá, Colombia; ^10^ School of Medicine and Health Sciences, Universidad Del Rosario, Bogotá, Colombia, urosario.edu.co; ^11^ Javeriana Oncology Center, Hospital Universitario San Ignacio, Bogotá, Colombia, husi.org.co

**Keywords:** immune checkpoint inhibitors, immune-related adverse events, immunotherapy, Latin America, melanoma, real-world evidence

## Abstract

**Background:**

Real‐world evidence on immune checkpoint inhibitors (ICIs) in Latin American patients with melanoma remains limited, particularly for acral and mucosal subtypes. We evaluated survival, radiologic response, immune‐related adverse events (irAEs), and exploratory factors associated with outcomes in a Colombian cohort.

**Methods:**

This single‐center retrospective cohort included consecutive patients with histologically confirmed melanoma who received ≥ 1 ICI dose between February 2017 and November 2022. Patients were classified as adjuvant‐ or palliative‐intent. rwOS and irAEs were assessed in the overall cohort; rwDFS in the adjuvant‐intent cohort; and rwPFS and best radiologic response in the palliative‐intent cohort. Survival was estimated using Kaplan–Meier methods, with median follow‐up calculated by reverse Kaplan–Meier. Primary Cox proportional hazards analyses of rwPFS and rwOS were restricted to the palliative‐intent cohort.

**Results:**

Fifty‐seven patients were included: 20 received adjuvant‐ and 37 palliative‐intent therapy. Median age was 65 years (IQR, 54–76); acral and mucosal melanoma accounted for 26 (45.6%) and 11 (19.3%) cases, respectively. Median follow‐up was 32.3 months (95% CI, 25.9–42.3). In the adjuvant‐intent cohort, median rwDFS and rwOS were 23.3 months (95% CI, 4.8–not reached) and 33.0 months (95% CI, 30.1–not reached), respectively. In the palliative‐intent cohort, median rwPFS and rwOS were 8.5 months (95% CI, 6.0–24.1) and 26.0 months (95% CI, 17.9–not reached), respectively. Among 31 response‐evaluable patients, ORR was 38.7% (95% CI, 23.7–56.2) and DCR was 54.8% (95% CI, 37.8–70.8). IrAEs were documented in 31 patients (54.4%); five had Grade 3 events, and no Grade 4 or five irAEs were documented. In adjusted models, ECOG performance status and melanoma subtype were not significantly associated with rwPFS or rwOS.

**Conclusions:**

ICIs demonstrated antitumor activity in this Colombian real‐world cohort, including patients with acral and mucosal melanoma, although survival estimates were numerically lower than those reported in selected pivotal trials. Documented toxicity was predominantly low grade. Exploratory analyses suggested more favorable outcomes in patients with conventional cutaneous melanoma.

## 1. Introduction

Melanoma is a malignant neoplasm arising from melanocytes and may occur in cutaneous, mucosal, or ocular sites [1]. According to GLOBOCAN 2022, an estimated 3,31,722 new melanoma cases and 58,667 melanoma‐related deaths occurred worldwide. Latin America accounts for approximately 20,291 new cases annually, with age‐standardized incidence and mortality rates of 2.4 and 0.65 per 1,00,000 population, respectively. In Colombia, the corresponding rates are 2.6 and 0.66 per 1,00,000 population, respectively [2, 3].

Immune checkpoint inhibitors (ICIs) have changed melanoma management by improving long‐term survival and producing durable responses in a subset of patients. These agents enhance antitumor immunity by inhibiting regulatory pathways such as cytotoxic T‐lymphocyte‐associated protein 4 and programmed cell death protein one or its ligand [4, 5]. However, clinical outcomes vary across melanoma subtypes. Acral and mucosal melanomas have distinct biological and genomic characteristics and are underrepresented in pivotal clinical trials, limiting the applicability of trial‐derived estimates to populations in which these subtypes are more prevalent. ICIs are also associated with immune‐related adverse events (irAEs), which may involve multiple organ systems and, in some cases, require corticosteroids, hormone‐replacement therapy, hospitalization, treatment interruption, or permanent discontinuation [5, 6]. Real‐world studies are therefore needed to characterize both clinical outcomes and toxicity in broader patient populations.

Evidence from Latin America remains limited and heterogeneous. A Peruvian study described clinicopathologic characteristics, BRAF status, response patterns, and survival outcomes in patients with advanced acral melanoma treated with immunotherapy [7]. A Brazilian cohort evaluated clinical and molecular factors associated with response and survival among patients with advanced melanoma receiving ICIs [8]. In Colombia, a retrospective cohort from Cali characterized survival outcomes in patients with mucosal melanoma [9]. Although these studies underscore the relevance of acral and mucosal melanoma in the region, the available evidence remains fragmented by melanoma subtype, treatment setting, and outcome assessed. Few Latin American cohorts have jointly evaluated survival, radiologic response, and immune‐related toxicity across both adjuvant‐ and palliative‐intent settings.

Differences in access to specialized oncology services, molecular testing, treatment authorization, and continuity of care may influence treatment delivery and the completeness of real‐world outcome assessment in Latin American settings [10]. In Colombia, oncology services remain geographically concentrated, and relatively few institutions provide the full range of cancer diagnostic and therapeutic services [11]. However, the effects of these healthcare‐system characteristics on individual patient outcomes cannot be assumed without direct patient‐level evaluation.

The primary objective of this retrospective cohort study was to estimate real‐world survival outcomes among patients with melanoma treated with ICIs at a tertiary cancer center in Colombia, separately for adjuvant‐ and palliative‐intent treatment. Secondary objectives were to describe the best documented radiologic response in the palliative‐intent cohort and characterize the frequency and maximum grade of irAEs. We also explored associations between selected clinical and treatment‐related characteristics and survival outcomes in the palliative‐intent cohort.

## 2. Methods

### 2.1. Study Design and Participants

We conducted a single‐center retrospective cohort study at Hospital Universitario San Ignacio, a tertiary referral cancer center affiliated with Pontificia Universidad Javeriana in Bogotá, Colombia. Consecutive patients with histologically confirmed melanoma who received at least one dose of ICI between February 27, 2017, and November 4, 2022, were eligible. Follow‐up was available through May 18, 2023.

ICI regimens included nivolumab, pembrolizumab, and nivolumab plus ipilimumab. Patients without histologic confirmation and those for whom an ICI was prescribed but never administered were excluded. No formal sample‐size calculation was performed because all consecutive eligible patients treated during the study period were included. Patients were classified according to treatment intent at ICI initiation. Adjuvant intent was defined as ICI administration after complete surgical resection, with no evidence of residual or measurable disease. Palliative intent was defined as ICI administration for metastatic disease, unresectable locoregional disease, or recurrent in‐transit, nodal, or distant disease. Patients initially diagnosed with earlier‐stage melanoma who subsequently developed unresectable or metastatic recurrence were classified in the palliative‐intent cohort.

This study is reported in accordance with the Strengthening the Reporting of Observational Studies in Epidemiology (STROBE) statement for cohort studies [12]. The completed STROBE checklist is provided as Supporting Information.

### 2.2. Data Collection and Variables

Demographic, clinical, pathological, molecular, treatment‐related, healthcare‐system, and safety data were retrospectively extracted from electronic medical records and entered into Research Electronic Data Capture (REDCap). Variables included age, sex, comorbidities, ECOG performance status, health insurance regimen, melanoma subtype, stage at diagnosis, LDH, central nervous system and hepatic involvement, BRAF mutation status, previous cancer‐directed treatments, treatment intent and line, ICI regimen, the recorded ICI formulation/order date, first administration date, radiologic response, recurrence or progression, death, and last documented follow‐up.

Stage at diagnosis was recorded as documented in the medical record. Because the American Joint Committee on Cancer staging edition was not consistently available, patients were not retrospectively restaged. BRAF status was recorded when available, although the molecular testing platform was not consistently documented. NRAS and KIT status, number of brain metastases or metastatic sites, and formal tumor‐burden measurements were not consistently available.

The formulation‐to‐administration interval was defined as the number of calendar days between the recorded ICI formulation/order date and first administration. It was examined as an exploratory, descriptive measure of the interval between the documented treatment order and treatment initiation. Dates of insurer authorization, pharmacy dispensing, and drug compounding were not separately available; therefore, this interval was not interpreted as a direct measure of any specific administrative process, diagnostic delay, or the broader pathway from diagnosis to treatment.

### 2.3. Radiologic Response Assessment

Radiologic response was assessed retrospectively using routine radiology reports obtained after ICI initiation and before subsequent systemic therapy. Imaging modality and timing followed routine clinical practice and included computed tomography, magnetic resonance imaging, or positron emission tomography, as clinically indicated.

Best documented response was classified as complete response, partial response, stable disease, or progressive disease based on the treating radiologist’s report, generally using RECIST 1.1 terminology when applicable. No blinded central review or retrospective image remeasurement was performed. ORR was defined as the proportion of response‐evaluable patients with complete or partial response, and DCR as the proportion with complete response, partial response, or stable disease. Patients without a valid posttreatment radiologic assessment were considered not evaluable and excluded from response denominators.

### 2.4. IrAEs

IrAEs were identified from clinical notes, laboratory findings, diagnostic assessments, hospitalizations, corticosteroid or hormone‐replacement therapy, and treatment modifications. Events were categorized by organ system and graded according to Common Terminology Criteria for Adverse Events Version 5.0 when sufficient information was available. When not formally documented, grade was assigned retrospectively only when supported by available clinical information; otherwise, events were classified as nonclassifiable. For patients with multiple irAEs, the highest documented grade was used to summarize maximum toxicity. Organ‐ and event‐specific categories were not mutually exclusive. IrAE occurrence was considered a postbaseline exposure.

### 2.5. Statistical Analysis

Categorical variables were summarized as frequencies and percentages, and continuous variables as medians and interquartile ranges (IQRs). Missing data were reported descriptively and were not imputed.

Survival distributions were estimated using the Kaplan–Meier method and compared using two‐sided log‐rank tests. Median follow‐up was estimated using the reverse Kaplan–Meier method. Median survival and survival probabilities at 1, 2, and 3 years were reported with 95% CIs. ORR and DCR were calculated among response‐evaluable patients, with 95% CIs estimated using the Wilson score method.

Primary Cox proportional hazards regression analyses were restricted to the palliative‐intent cohort. Univariable models evaluated age per 10‐year increase, sex, ECOG performance status (≥ 2 vs 0–1), melanoma subtype (conventional cutaneous vs all other subtypes), BRAF mutation status, LDH elevation, central nervous system involvement, hepatic involvement, treatment line, ICI regimen, and irAE occurrence. Primary multivariable models included two clinically relevant baseline variables: ECOG performance status and melanoma subtype (conventional cutaneous vs all other subtypes). No automated variable‐selection procedure was used.

Two additional analyses were exploratory. The first was restricted to patients with conventional cutaneous or acral melanoma and evaluated melanoma subtype adjusted for ECOG performance status. The second included ECOG performance status and irAE occurrence, with irAE modeled as a fixed exposure. Because this approach did not account for time to irAE onset, estimates were considered susceptible to immortal‐time bias and were not interpreted causally. In addition, a post hoc 6‐month landmark sensitivity analysis was performed among patients with ascertainable irAE timing. The landmark was selected because it approximated the median documented time to irAE onset (5.6 months) and represented an early‐treatment interval. Patients with irAE onset on or before 6 months were classified as having an early irAE; patients without an irAE or with documented onset after 6 months were classified as having no early irAE. Patients with an irAE but no documented onset date were excluded, as were patients with an event or censoring on or before the landmark. Follow‐up time was reset at 6 months. RwDFS and rwOS were assessed in the adjuvant‐intent cohort, rwPFS and rwOS in the palliative‐intent cohort, and overall rwOS using a Cox model stratified by treatment intent. Landmark Cox models were unadjusted because of sparse events, and hazard ratios (HRs) were not estimated when one exposure group had no events. Kaplan–Meier estimates and log‐rank tests were also calculated.

HRs and 95% CIs were estimated using Cox regression. The proportional hazards assumption was assessed using scaled Schoenfeld residuals, individual and global tests, and visual inspection of residual plots. Multicollinearity was evaluated using variance inflation factors.

Complete‐case analysis was used for each model, with sample size varying according to variable availability. Baseline characteristics of the palliative‐intent cohort were also summarized descriptively according to irAE occurrence; no formal hypothesis testing was performed for this postbaseline stratification. No adjustment for multiple comparisons was performed because regression and subgroup analyses were exploratory. All statistical tests were two‐sided, and *p* values  < 0.05 were considered statistically significant. Analyses were performed in Python within Google Colaboratory using pandas and NumPy for data management and descriptive statistics, statsmodels for Wilson 95% CIs, lifelines for Kaplan–Meier, reverse Kaplan–Meier, primary Cox regression, and Schoenfeld residuals, SciPy and NumPy for the landmark sensitivity analysis, and Matplotlib for figures.

### 2.6. Ethical Considerations

The study was conducted in accordance with the Declaration of Helsinki and was approved by the Institutional Research and Ethics Committee of the Faculty of Medicine of Pontificia Universidad Javeriana and Hospital Universitario San Ignacio, Bogotá, Colombia.

Approval was granted on July 8, 2021, according to Act No. 12/2021, under study reference 2021/56. The formal approval communication was issued on July 13, 2021, under institutional code FM‐CIE‐0673‐21. The study was classified as minimal risk, and the requirement for informed consent was waived because routinely collected, de‐identified clinical data were analyzed without direct patient intervention.

## 3. Results

### 3.1. Patient Characteristics

The analytic cohort included 57 patients, of whom 20 (35.1%) received adjuvant‐intent ICIs and 37 (64.9%) received palliative‐intent ICIs. Median age was 65 years (IQR, 54–76), 31 patients (54.4%) were female, and 51 (89.5%) had an ECOG performance status of 0–1. Acral melanoma was the most frequent subtype (26/57, 45.6%), followed by cutaneous (17/57, 29.8%) and mucosal melanoma (11/57, 19.3%).

At initial melanoma diagnosis, 1 patient (1.8%) had Stage I disease, 5 (8.8%) had Stage II disease, 31 (54.4%) had Stage III disease, 18 (31.6%) had Stage IV disease, and 2 (3.5%) had unknown stage. All patients in the adjuvant‐intent cohort had Stage III disease at diagnosis. Among patients receiving palliative‐intent treatment, 18 of 37 (48.6%) had Stage IV disease at diagnosis, whereas 19 (51.4%) had initially presented with earlier‐stage or unknown‐stage disease and subsequently received ICIs after unresectable recurrence or progression. Of the 18 patients with Stage IV disease at diagnosis, 11 received first‐line and 7 received second‐line palliative ICIs.

Twenty‐seven of 37 palliative‐intent patients (73.0%) received ICIs as first‐line therapy, and 10 (27.0%) as second‐line therapy. BRAF status was available in 50 patients (87.7%), of whom 10 (17.5%) had BRAF‐mutated melanoma. All 10 patients with a documented BRAF mutation had V600‐class mutations. LDH was unavailable in 23 (40.4%). Central nervous system and hepatic involvement were documented in 3 (5.3%) and 6 patients (10.5%), respectively, all within the palliative‐intent cohort. Baseline clinical, molecular, healthcare‐system, and treatment characteristics are presented in Table 1. Additional histopathologic and prior‐treatment data are provided in Supporting Table S1, and characteristics according to palliative treatment line are shown in Supporting Table S2.

**TABLE 1:** Baseline characteristics stratified by treatment intent at immune checkpoint inhibitor initiation.

Characteristic	Total *n* = 57	Adjuvant *n* = 20	Palliative *n* = 37
Age, years, median (IQR)	65.0 (54.0–76.0)	66.0 (53.8–80.3)	63.0 (54.0–75.0)
Sex, *n* (%)			
Female	31 (54.4)	9 (45.0)	22 (59.5)
Male	26 (45.6)	11 (55.0)	15 (40.5)
Health insurance regimen, *n* (%)			
Contributory	51 (89.5)	17 (85.0)	34 (91.9)
Subsidized	6 (10.5)	3 (15.0)	3 (8.1)
ECOG performance status, *n* (%)			
0	15 (26.3)	8 (40.0)	7 (18.9)
1	36 (63.2)	11 (55.0)	25 (67.6)
2	5 (8.8)	1 (5.0)	4 (10.8)
3	1 (1.8)	0 (0.0)	1 (2.7)
Stage at initial diagnosis, *n* (%)			
I	1 (1.8)	0 (0.0)	1 (2.7)
II	5 (8.8)	0 (0.0)	5 (13.5)
III	31 (54.4)	20 (100.0)	11 (29.7)
IV	18 (31.6)	0 (0.0)	18 (48.6)
Unknown	2 (3.5)	0 (0.0)	2 (5.4)
Melanoma subtype, *n* (%)			
Cutaneous	17 (29.8)	5 (25.0)	12 (32.4)
Acral	26 (45.6)	14 (70.0)	12 (32.4)
Mucosal	11 (19.3)	1 (5.0)	10 (27.0)
Uveal	1 (1.8)	0 (0.0)	1 (2.7)
Unknown primary	2 (3.5)	0 (0.0)	2 (5.4)
BRAF status, *n* (%)			
Mutated	10 (17.5)	3 (15.0)	7 (18.9)
Wild type	40 (70.2)	15 (75.0)	25 (67.6)
Not reported	7 (12.3)	2 (10.0)	5 (13.5)
LDH value, U/L, median (IQR)	211.5 (158.8–378.8)	166.5 (152.8–206.0)	221.5 (178.8–419.0)
LDH category, *n* (%)			
Within range (140–271 U/L)	21 (36.8)	8 (40.0)	13 (35.1)
Elevated (> 271 U/L)	11 (19.3)	2 (10.0)	9 (24.3)
Below range (< 140 U/L)	2 (3.5)	0 (0.0)	2 (5.4)
Not available	23 (40.4)	10 (50.0)	13 (35.1)
CNS involvement, *n* (%)	3 (5.3)	0 (0.0)	3 (8.1)
Hepatic involvement, *n* (%)	6 (10.5)	0 (0.0)	6 (16.2)
Prior oncologic treatment, *n* (%)	48 (84.2)	19 (95.0)	29 (78.4)
Treatment line, *n* (%)			
Adjuvant	20 (35.1)	20 (100.0)	0 (0.0)
First‐line palliative	27 (47.4)	0 (0.0)	27 (73.0)
Second‐line palliative	10 (17.5)	0 (0.0)	10 (27.0)
ICI regimen, *n* (%)			
Pembrolizumab	23 (40.4)	4 (20.0)	19 (51.4)
Nivolumab	21 (36.8)	16 (80.0)	5 (13.5)
Nivolumab plus ipilimumab	13 (22.8)	0 (0.0)	13 (35.1)

*Note:* Stage refers to the disease stage at initial melanoma diagnosis. Treatment groups were defined according to treatment intent at ICI initiation. Percentages were calculated using the total number of patients in each treatment‐intent group as the denominator. LDH medians were calculated among patients with available LDH measurements: 34 patients overall, 10 in the adjuvant cohort, and 24 in the palliative cohort. Any prior oncologic treatment refers to cancer‐directed treatment received before ICI initiation. BRAF, B‐Raf proto‐oncogene; LDH, lactate dehydrogenase.

Abbreviations: CNS, central nervous system; ECOG, Eastern Cooperative Oncology Group; ICI, immune checkpoint inhibitor; IQR, interquartile range.

The median interval from the recorded ICI formulation/order date to first administration was 15 days (IQR, 10–22) overall, 12 days (IQR, 9–20.3) in the adjuvant‐intent cohort, and 17 days (IQR, 11–25) in the palliative‐intent cohort.

### 3.2. Radiologic Response in the Palliative‐Intent Cohort

Of the 37 patients receiving palliative‐intent ICIs, 31 were evaluable for radiologic response, and 6 had no valid posttreatment response assessment. Among evaluable patients, 6 achieved a complete response, 6 a partial response, 5 had stable disease, and 14 had progressive disease. The ORR was 38.7% (12/31; 95% CI, 23.7–56.2), and the DCR was 54.8% (17/31; 95% CI, 37.8–70.8).

Among patients receiving first‐line ICIs, 22 of 27 were evaluable. The ORR was 40.9% (9/22; 95% CI, 23.3–61.3), and the DCR was 54.5% (12/22; 95% CI, 34.7–73.1). Among patients receiving second‐line ICIs, 9 of 10 were evaluable; the ORR was 33.3% (3/9; 95% CI, 12.1–64.6), and the DCR was 55.6% (5/9; 95% CI, 26.7–81.1). Response outcomes according to treatment line and ICI regimen are presented in Supporting Table S3.

In exploratory analyses of the major melanoma subtypes, ORR was 54.5% (6/11; 95% CI, 28.0–78.7) in cutaneous melanoma, 11.1% (1/9; 95% CI, 2.0–43.5) in acral melanoma, and 50.0% (4/8; 95% CI, 21.5–78.5) in mucosal melanoma. These estimates were imprecise because of the small subgroup sizes.

### 3.3. Survival Outcomes and Exploratory Analyses

Median follow‐up, estimated using the reverse Kaplan–Meier method, was 32.3 months (95% CI, 25.9–42.3). Subsequent systemic or local anticancer treatments administered after recurrence or progression, including in the adjuvant‐intent cohort, were not systematically captured in either cohort and could not be summarized, limiting interpretation of rwOS.

Ten rwDFS events and 7 deaths occurred. Median rwDFS was 23.3 months (95% CI, 4.8–not reached [NR]), and median rwOS was 33.0 months (95% CI, 30.1–NR). Estimated rwDFS rates at 1, 2, and 3 years were 60.0%, 48.5%, and 48.5%, respectively; rwOS rates were 90.0%, 78.3%, and 44.1% Figure 1A,B. Among patients in the adjuvant‐intent cohort, median rwDFS was 12.4 months in those with acral melanoma versus NR in those with cutaneous melanoma (HR, 1.86; 95% CI, 0.39–8.84; log‐rank *p* = 0.427). Median rwOS was 33.0 versus 31.9 months, respectively (HR, 2.80; 95% CI, 0.33–23.51; log‐rank *p* = 0.323). Estimates were imprecise because of small subgroup sizes and few events (Supporting Figures S1A,B).

**FIGURE 1:**
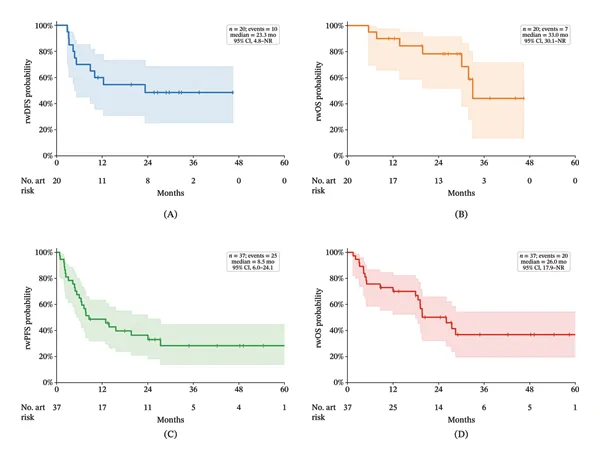
Kaplan–Meier estimates of survival outcomes according to treatment intent: Kaplan–Meier estimates of (A) real‐world disease‐free survival (rwDFS) and (B) real‐world overall survival (rwOS) in the adjuvant‐intent cohort, and (C) real‐world progression‐free survival (rwPFS) and (D) rwOS in the palliative‐intent cohort. Shaded areas represent 95% confidence intervals, vertical tick marks indicate censored observations, and the numbers below each panel indicate patients at risk. All panels are displayed on a common time scale from 0 to 60 months.

Twenty‐five rwPFS events and 20 deaths occurred. Median rwPFS was 8.5 months (95% CI, 6.0–24.1), and median rwOS was 26.0 months (95% CI, 17.9–NR). Estimated rwPFS rates at 1, 2, and 3 years were 48.6%, 36.4%, and 28.3%, respectively; rwOS rates were 70.1%, 50.2%, and 36.8% Figure 1C,D. Among patients with cutaneous or acral melanoma, median rwPFS was 15.6 months for cutaneous versus 6.0 months for acral melanoma (HR, 0.33; 95% CI, 0.12–0.92; log‐rank *p* = 0.026). Median rwOS was NR versus 12.0 months, respectively (HR, 0.31; 95% CI, 0.09–1.00; log‐rank *p* = 0.039) (Supporting Figures S2A,B).

In univariable analyses, none of the prespecified baseline demographic, clinical, molecular, or treatment‐related variables was significantly associated with rwPFS or rwOS. In the primary multivariable models, ECOG performance status ≥ 2 was not significantly associated with rwPFS (adjusted HR, 1.99; 95% CI, 0.67–5.93; *p* = 0.217) or rwOS (adjusted HR, 2.25; 95% CI, 0.74–6.79; *p* = 0.151). Conventional cutaneous melanoma, compared with all other melanoma subtypes, was associated with numerically lower hazards of progression or death (adjusted HR, 0.47; 95% CI, 0.19–1.20; *p* = 0.115) and death (adjusted HR, 0.42; 95% CI, 0.14–1.27; *p* = 0.124), although neither association reached statistical significance Table 2. There was no evidence of violation of the proportional hazards assumption (global Schoenfeld test: rwPFS, *χ* ^2^ = 0.06, 2 df, *p* = 0.972; rwOS, *χ* ^2^ = 1.03, 2 df, *p* = 0.596), and variance inflation factors were 1.07 for both covariates.

**TABLE 2:** Univariable and multivariable Cox proportional hazards analyses in the palliative‐intent cohort.

Variable	Univariable rwPFS HR (95% CI), *p*	Multivariable rwPFS HR (95% CI), *p*	Univariable rwOS HR (95% CI), *p*	Multivariable rwOS HR (95% CI), *p*
Age, per 10‐year increase	0.97 (0.75–1.26), 0.842	—	1.08 (0.79–1.47), 0.621	—
Female vs male	1.02 (0.45–2.32), 0.964	—	0.70 (0.28–1.74), 0.437	—
ECOG ≥ 2 vs 0–1	1.75 (0.60–5.15), 0.309	1.99 (0.67–5.93), 0.217	2.15 (0.71–6.48), 0.174	2.25 (0.74–6.79), 0.151
Conventional cutaneous vs other melanoma subtypes	0.50 (0.20–1.26), 0.143	0.47 (0.19–1.20), 0.115	0.43 (0.14–1.30), 0.136	0.42 (0.14–1.27), 0.124
BRAF mutated vs wild type	0.59 (0.17–2.03), 0.405	—	0.95 (0.27–3.37), 0.941	—
Elevated vs nonelevated LDH	2.04 (0.78–5.35), 0.147	—	2.04 (0.72–5.75), 0.180	—
CNS involvement: yes vs no	0.32 (0.04–2.37), 0.265	—	0.49 (0.07–3.69), 0.489	—
Hepatic involvement: yes vs no	1.59 (0.63–3.99), 0.325	—	1.45 (0.48–4.36), 0.512	—
Second‐line vs first‐line ICI	1.08 (0.45–2.59), 0.864	—	0.92 (0.33–2.55), 0.877	—
Nivolumab plus ipilimumab vs anti‐PD‐1 monotherapy	1.17 (0.51–2.68), 0.715	—	1.46 (0.58–3.67), 0.419	—
IrAE yes vs no	0.41 (0.18–0.91), 0.029	—	0.27 (0.11–0.69), 0.006	—

*Note:* The primary multivariable model included ECOG performance status and melanoma subtype (conventional cutaneous vs other melanoma subtypes). An em dash (—) indicates that a variable was not included in the primary multivariable model. Analyses were based on complete cases; BRAF analyses included 32 patients and LDH analyses included 24 patients. IrAE occurrence was evaluated as an exploratory fixed postbaseline exposure and was not included in the primary model because of potential immortal‐time bias. The primary models showed no evidence of proportional hazards violation (global Schoenfeld test: rwPFS *p* = 0.972; rwOS *p* = 0.596). The univariable nivolumab plus ipilimumab versus anti‐PD‐1 monotherapy model for rwOS showed possible nonproportionality (Schoenfeld *p* = 0.019). LDH, lactate dehydrogenase.

Abbreviations: CI, confidence interval; CNS, central nervous system; ECOG, Eastern Cooperative Oncology Group; HR, hazard ratio; ICI, immune checkpoint inhibitor; irAE, immune‐related adverse event; rwOS, real‐world overall survival; rwPFS, real‐world progression‐free survival.

In the exploratory analysis restricted to cutaneous and acral melanoma, cutaneous melanoma was associated with a lower hazard of progression or death after adjustment for ECOG performance status (adjusted HR, 0.34; 95% CI, 0.12–0.92; *p* = 0.034), whereas the association with rwOS did not reach statistical significance (adjusted HR, 0.32; 95% CI, 0.10–1.06; *p* = 0.062) (Supporting Table S6).

### 3.4. IrAEs

At least one irAE was documented in 31 of 57 patients (54.4%), including 11 of 20 patients (55.0%) in the adjuvant‐intent cohort and 20 of 37 (54.1%) in the palliative‐intent cohort. Among these 31 patients, the maximum documented grade was 1–2 in 21 (67.7%), Grade 3 in 5 (16.1%), and nonclassifiable or undocumented in 5 (16.1%). No Grade 4 or five irAEs were documented.

Endocrine irAEs were the most frequently documented, occurring in 7 adjuvant‐intent patients (35.0%) and 15 palliative‐intent patients (40.5%). Gastrointestinal irAEs occurred in 4 (20.0%) and 7 patients (18.9%), respectively. Primary adrenal insufficiency was the most frequent specific event, documented in 4 adjuvant‐intent patients (20.0%) and 6 palliative‐intent patients (16.2%). Other recurrent events included hypothyroidism, transaminase elevation without hyperbilirubinemia, hypophysitis, and diarrhea or colitis (Supporting Table S4). Treatment discontinuation specifically attributable to an irAE was not systematically documented and could not be reliably summarized.

In exploratory survival analyses, median rwDFS was NR among patients with irAEs versus 5.2 months among those without irAEs in the adjuvant‐intent cohort (log‐rank *p* = 0.002). Median rwOS was NR versus 30.1 months, respectively (log‐rank *p* = 0.031). In the palliative‐intent cohort, median rwPFS was 15.6 versus 5.3 months (log‐rank *p* = 0.024), and median rwOS was NR versus 12.0 months, respectively (log‐rank *p* = 0.004) (Supporting Figure S3A–D). Baseline characteristics of the palliative‐intent cohort according to irAE occurrence are presented descriptively in Supporting Table S5.

In the palliative‐intent cohort, univariable Cox analyses showed lower hazards of progression or death (HR, 0.41; 95% CI, 0.18–0.91; *p* = 0.029) and death (HR, 0.27; 95% CI, 0.11–0.69; *p* = 0.006) among patients with irAEs. After adjustment for ECOG performance status, irAE occurrence remained associated with lower hazards of progression or death (adjusted HR, 0.36; 95% CI, 0.16–0.83; *p* = 0.016) and death (adjusted HR, 0.20; 95% CI, 0.07–0.56; *p* = 0.002) (Supporting Table S6). Because irAE occurrence was modeled as a fixed postbaseline exposure, these estimates are susceptible to immortal‐time bias and should not be interpreted causally.

The date of irAE onset was documented for 15 of 31 patients with an irAE (48.4%), with a median time to onset of 5.6 months (IQR, 2.7–10.0). At the 6‐month landmark, 32 patients were evaluable for overall rwOS: 8 had experienced an irAE by the landmark, and 24 had not; 12 subsequent deaths occurred. In a Cox model stratified by treatment intent, there was no evidence of an association between an irAE by 6 months and subsequent rwOS (HR, 0.81; 95% CI, 0.17–3.71; *p* = 0.781; stratified log‐rank *p* = 0.781). In the palliative‐intent cohort, 16 patients were evaluable for rwPFS (5 with an irAE by 6 months and 11 without; 7 subsequent events) and 18 for rwOS (5 and 13, respectively; 7 subsequent deaths). The corresponding unadjusted HRs were 1.38 (95% CI, 0.25–7.53; *p* = 0.709) for rwPFS and 1.84 (95% CI, 0.35–9.69; *p* = 0.470) for rwOS. In the adjuvant‐intent cohort, 10 patients were evaluable for rwDFS and 14 for rwOS; no subsequent events occurred among the 3 patients with an irAE by the landmark, precluding stable HR estimation. Because 16 patients with an irAE lacked an onset date and the landmark risk sets contained few events, these estimates were highly imprecise and the analysis was inconclusive.

## 4. Discussion

In this single‐center retrospective cohort, we described real‐world outcomes among 57 Colombian patients with melanoma treated with ICIs, including a high representation of acral and mucosal melanoma. In the adjuvant‐intent cohort, median rwDFS and rwOS were 23.3 and 33.0 months, respectively, while in the palliative‐intent cohort, median rwPFS and rwOS were 8.5 and 26.0 months, with an ORR of 38.7% among response‐evaluable patients. irAEs were documented in 54.4% of patients and were predominantly Grade 1–2. These findings provide descriptive real‐world estimates from Colombia in a population with a substantial representation of melanoma subtypes that remain underrepresented in pivotal ICI trials.

The distribution of melanoma subtypes is central to interpreting these findings. Acral and mucosal melanomas accounted for 26 of 57 (45.6%) and 11 of 57 patients (19.3%), respectively, and acral melanoma represented 14 of 20 patients (70.0%) in the adjuvant‐intent group. These subtypes differ biologically and genomically from ultraviolet‐associated conventional cutaneous melanoma and have been underrepresented in pivotal ICI trials [13]. Their substantial representation in our cohort is consistent with reports from Peru, Brazil, Colombia, and other Latin American settings describing a relatively high burden of acral, mucosal, and advanced‐stage melanoma [7–9, 14–19]. Our study extends this regional evidence by evaluating adjuvant and palliative outcomes, radiologic response, treatment‐delivery timing, and immune‐related toxicity within the same real‐world cohort. Consistent with their distinct biology, acral and mucosal melanomas have shown lower objective response rates and shorter survival with immune checkpoint inhibition than cutaneous melanoma in pooled trial and real‐world analyses [13], a pattern reflected in the lower acral response rate observed in our palliative cohort.

In the adjuvant‐intent cohort, median rwDFS was 23.3 months, and the estimated 3‐year rwDFS rate was 48.5%; however, these estimates were imprecise because only 20 patients were included, and few remained at risk at later time points. CheckMate 238 and KEYNOTE‐054 reported 5‐year recurrence‐free survival rates of approximately 50% and 55.4% with adjuvant nivolumab and pembrolizumab, respectively [20, 21]. A Brazilian real‐world cohort similarly highlighted challenges in adjuvant treatment delivery, with only 44.6% of patients completing 1 year of adjuvant treatment [22]. Direct comparisons are not appropriate because the present cohort was predominantly composed of acral melanoma. These findings should therefore be viewed as descriptive regional estimates rather than evidence of inferior or equivalent effectiveness relative to randomized trials.

In the palliative‐intent cohort, median rwPFS was 8.5 months, median rwOS was 26.0 months, and ORR was 38.7%. These outcomes fall within the range reported across ICI‐based treatment studies in advanced melanoma, although registrational trials such as KEYNOTE‐006, CheckMate 067, and RELATIVITY‐047 enrolled more selected populations with protocol‐defined treatment and response assessment [23–25]. Our cohort differed from these trials in its substantial representation of acral and mucosal melanoma, inclusion of first‐ and second‐line treatment, heterogeneous ICI regimens, and incomplete characterization of LDH and metastatic burden. The observed outcomes therefore cannot be attributed specifically to ICI treatment or directly compared with randomized trials. Nevertheless, they are broadly consistent with pooled and real‐world evidence, suggesting lower ICI response rates in populations enriched for acral and mucosal melanoma [7, 13, 26].

The exploratory comparison between cutaneous and acral melanoma provides additional context. Among patients with cutaneous or acral melanoma in the palliative‐intent cohort, cutaneous melanoma was associated with longer rwPFS and lower hazard of progression or death after adjustment for ECOG performance status, whereas the association with rwOS did not reach statistical significance. This finding is biologically plausible and consistent with previous evidence of lower ICI responsiveness in acral melanoma [7, 13]. However, the subgroup analysis included few patients and events and was exploratory; therefore, it should be considered hypothesis‐generating rather than evidence of an independent prognostic effect. Similarly, differences in response according to treatment line should be interpreted cautiously given the small numbers and heterogeneous treatment exposure.

The treatment‐delivery interval provides an additional real‐world perspective. The median interval from the recorded ICI formulation/order date to first administration was 15 days (IQR, 10–22), with longer intervals in the palliative‐intent cohort than in the adjuvant‐intent cohort (17 days [IQR, 11–25] vs 12 days [IQR, 9–20.3]). This descriptive measure reflects the recorded interval between the treatment order and first administration. Because insurer‐authorization, pharmacy‐dispensing, and compounding dates were not separately available, it cannot identify delays attributable to any specific administrative step. In Colombia, ICIs may be financed through the national health system, with the applicable mechanism depending on the indication and current coverage rules [27]. Treatment delivery may nevertheless be influenced by insurer and provider administrative processes, geographic concentration of oncology services, and differences between care settings [10, 11, 28]. Across Latin America, access to ICIs remains heterogeneous, with availability and reimbursement varying between countries and healthcare systems [7, 28]. Our study did not collect sufficiently detailed patient‐level socioeconomic or access data to determine whether these factors influenced treatment delivery or outcomes. The predominance of patients in the contributory regime also limited meaningful comparisons by insurance status.

IrAEs were documented in 54.4% of patients, with most maximum documented grades being 1–2; five patients experienced Grade 3 events, and no Grade 4 or 5 events were documented. Endocrine events were the most frequently documented, followed by gastrointestinal events, consistent with the multisystem toxicity profile of ICIs [5, 6, 29]. Treatment discontinuation specifically attributable to an irAE was not systematically documented, limiting interpretation of the clinical consequences of toxicity. Patients with irAEs had longer Kaplan–Meier estimates of rwDFS, rwPFS, and rwOS, and irAE occurrence was associated with lower hazards of progression or death in exploratory fixed‐exposure Cox analyses. However, the post hoc 6‐month landmark sensitivity analysis did not provide evidence of an association between early‐irAE occurrence and subsequent survival. All landmark estimates were highly imprecise, and the adjuvant‐intent HRs could not be estimated because no events occurred in the small early‐irAE group. These findings are compatible with immortal‐time bias contributing to the fixed‐exposure associations, but missing onset dates and sparse events preclude firm conclusions. The irAE–survival association should therefore be considered exploratory, inconclusive, and noncausal.

This study has several strengths, including consecutive inclusion of patients treated in routine practice, separate evaluation of adjuvant‐ and palliative‐intent cohorts, explicit characterization of missing data, and integrated assessment of survival, radiologic response, toxicity, and treatment‐delivery intervals. These features provide clinically relevant information from a Latin American setting in which acral and mucosal melanoma constitute a substantial proportion of the treated population. The study also has important limitations. Its retrospective single‐center design and small sample size limit precision and generalizability; the absence of a comparator group precludes causal inference; imaging schedules and response assessment were not standardized and lacked blinded central review; molecular and metastatic‐burden data were incomplete; and postrecurrence or postprogression systemic or local anticancer treatments were not systematically captured in either cohort, limiting interpretation of rwOS. In addition, treatment discontinuation attributable to irAEs and patient‐level socioeconomic or access factors were incompletely documented. Subgroup and regression analyses were exploratory, and no adjustment for multiple comparisons was performed. Fixed‐exposure analyses according to irAE occurrence were susceptible to immortal‐time bias. Although a 6‐month landmark sensitivity analysis was added, onset dates were missing for 16 of 31 patients with an irAE, and the resulting risk sets and event counts were small; therefore, this analysis could only partly address the bias and yielded imprecise estimates. Overall, these findings should be viewed as hypothesis‐generating real‐world estimates that complement, rather than replace, evidence from prospective clinical trials and larger multicenter Latin American cohorts.

## 5. Conclusion

This study provides real‐world evidence of antitumor activity with ICIs in Colombian patients with melanoma, including substantial representation of acral and mucosal subtypes. Survival estimates were numerically lower than those reported in selected pivotal trials, potentially reflecting the heterogeneous real‐world population and differences in melanoma subtype, treatment setting, and baseline disease characteristics. Documented immune‐related toxicity was predominantly low grade, with no Grade 4 or 5 events documented. Exploratory analyses suggested more favorable outcomes among patients with conventional cutaneous melanoma. Associations between irAE occurrence and survival remain exploratory and noncausal because fixed‐exposure analyses were vulnerable to immortal‐time bias and the landmark sensitivity analysis was limited by missing onset dates and sparse events.

## Funding

This research received no specific grant from any funding agency in the public, commercial, or not‐for‐profit sectors.

## Consent

The authors have nothing to report.

## Conflicts of Interest

The authors declare no conflicts of interest.

## Supporting Information

Additional supporting information can be found online in the Supporting Information section.

## Supporting information


**Supporting Information** The following supporting materials accompany this article: Supporting Table S1. Detailed baseline, histologic, staging, molecular, and prior‐treatment characteristics, stratified by treatment intent. Supporting Table S2. Baseline clinical, molecular, and treatment characteristics of the palliative‐intent cohort, stratified by treatment line. Supporting Table S3. Best documented radiologic response, objective response rate, and disease control rate in the palliative‐intent cohort, according to treatment line and immune checkpoint inhibitor regimen. Supporting Table S4. Immune‐related adverse events according to treatment intent, organ system, specific event, and maximum grade. Supporting Table S5. Baseline characteristics of the palliative‐intent cohort according to immune‐related adverse event occurrence. Supporting Table S6. Exploratory multivariable Cox proportional hazards models in the palliative‐intent cohort. Supporting Figure S1. Kaplan–Meier estimates of real‐world disease‐free survival (*A*) and real‐world overall survival (*B*) in the adjuvant‐intent cohort, stratified by acral versus nonacral melanoma. Supporting Figure S2. Kaplan–Meier estimates of real‐world progression‐free survival (*A*) and real‐world overall survival (*B*) in the palliative‐intent cohort, restricted to patients with conventional cutaneous or acral melanoma. Supporting Figure S3. Kaplan–Meier estimates according to immune‐related adverse event occurrence: real‐world disease‐free survival (*A*) and real‐world overall survival (*B*) in the adjuvant‐intent cohort, and real‐world progression‐free survival (*C*) and real‐world overall survival (*D*) in the palliative‐intent cohort. Supporting File S1. Completed STROBE checklist for cohort studies.

## Data Availability

The data analyzed in this study are not publicly available because of patient confidentiality and institutional restrictions. Deidentified data may be obtained from the corresponding author upon reasonable request and subject to institutional approval.
