# Supplementary material for: Real‐World Outcomes of Immune Checkpoint Inhibitors in Melanoma, Including Acral and Mucosal Subtypes: A Retrospective Cohort Study From a Latin American Center
Source: J Skin Cancer. 2026 Sep 28;2026:3904810. doi: 10.1155/jskc/3904810 (PMC13617541; doi:10.1155/jskc/3904810)
Supplement: Supplementary file 1 [file JSKC-2026-3904810-s001.zip › Supplements.docx]

## Supplementary Materials

The following supplementary materials accompany this article:

**Supplementary Table S1.** Detailed baseline, histologic, staging, molecular, and prior-treatment characteristics, stratified by treatment intent.

**Supplementary Table S2.** Baseline clinical, molecular, and treatment characteristics of the palliative-intent cohort, stratified by treatment line.

**Supplementary Table S3.** Best documented radiologic response, objective response rate, and disease control rate in the palliative-intent cohort, according to treatment line and immune checkpoint inhibitor regimen.

**Supplementary Table S4.** Immune-related adverse events according to treatment intent, organ system, specific event, and maximum grade.

**Supplementary Table S5.** Baseline characteristics of the palliative-intent cohort according to immune-related adverse event occurrence.

**Supplementary Table S6.** Exploratory multivariable Cox proportional hazards models in the palliative-intent cohort.

**Supplementary Figure S1.** Kaplan–Meier estimates of real-world disease-free survival (A) and real-world overall survival (B) in the adjuvant-intent cohort, stratified by acral versus non-acral melanoma.

**Supplementary Figure S2.** Kaplan–Meier estimates of real-world progression-free survival (A) and real-world overall survival (B) in the palliative-intent cohort, restricted to patients with conventional cutaneous or acral melanoma.

**Supplementary Figure S3.** Kaplan–Meier estimates according to immune-related adverse event occurrence: real-world disease-free survival (A) and real-world overall survival (B) in the adjuvant-intent cohort, and real-world progression-free survival (C) and real-world overall survival (D) in the palliative-intent cohort.

**Supplementary File S1.** Completed STROBE checklist for cohort studies.

| **Characteristic** | **Total n=57** | **Adjuvant n=20** | **Palliative n=37** |
| --- | --- | --- | --- |
| **Histologic subtype, n (%)** | | | |
| Nodular | 10 (17.5) | 2 (10.0) | 8 (21.6) |
| Acral lentiginous | 24 (42.1) | 13 (65.0) | 11 (29.7) |
| Not otherwise specified | 15 (26.3) | 3 (15.0) | 12 (32.4) |
| Superficial spreading | 7 (12.3) | 2 (10.0) | 5 (13.5) |
| Lentiginous | 1 (1.8) | 0 (0.0) | 1 (2.7) |
| **Detailed stage at initial diagnosis, n (%)** | | | |
| IB | 1 (1.8) | 0 (0.0) | 1 (2.7) |
| IIB | 1 (1.8) | 0 (0.0) | 1 (2.7) |
| IIC | 4 (7.0) | 0 (0.0) | 4 (10.8) |
| IIIA | 4 (7.0) | 1 (5.0) | 3 (8.1) |
| IIIB | 6 (10.5) | 2 (10.0) | 4 (10.8) |
| IIIC | 20 (35.1) | 16 (80.0) | 4 (10.8) |
| IIID | 1 (1.8) | 1 (5.0) | 0 (0.0) |
| IV | 17 (29.8) | 0 (0.0) | 17 (45.9) |
| IVB | 1 (1.8) | 0 (0.0) | 1 (2.7) |
| Unknown | 2 (3.5) | 0 (0.0) | 2 (5.4) |
| **Primary tumor location, n (%)** | | | |
| Lower limb | 25 (43.9) | 12 (60.0) | 13 (35.1) |
| Upper limb | 8 (14.0) | 4 (20.0) | 4 (10.8) |
| Head and neck | 4 (7.0) | 2 (10.0) | 2 (5.4) |
| Trunk | 5 (8.8) | 1 (5.0) | 4 (10.8) |
| Mucosal | 11 (19.3) | 1 (5.0) | 10 (27.0) |
| Cutaneous, unspecified | 1 (1.8) | 0 (0.0) | 1 (2.7) |
| Uveal | 1 (1.8) | 0 (0.0) | 1 (2.7) |
| Unknown primary | 2 (3.5) | 0 (0.0) | 2 (5.4) |
| **Prior oncologic treatment, n (%)** | | | |
| No | 9 (15.8) | 1 (5.0) | 8 (21.6) |
| Yes | 48 (84.2) | 19 (95.0) | 29 (78.4) |
| **Prior chemotherapy, n (%)** | | | |
| Not applicable | 9 (15.8) | 1 (5.0) | 8 (21.6) |
| No | 45 (78.9) | 19 (95.0) | 26 (70.3) |
| Yes | 3 (5.3) | 0 (0.0) | 3 (8.1) |
| **Prior radiotherapy, n (%)** | | | |
| Not applicable | 9 (15.8) | 1 (5.0) | 8 (21.6) |
| No | 34 (59.6) | 19 (95.0) | 15 (40.5) |
| Yes | 14 (24.6) | 0 (0.0) | 14 (37.8) |
| **Prior surgery, n (%)** | | | |
| Not applicable | 9 (15.8) | 1 (5.0) | 8 (21.6) |
| No | 7 (12.3) | 0 (0.0) | 7 (18.9) |
| Yes | 41 (71.9) | 19 (95.0) | 22 (59.5) |
| **Prior targeted therapy, n (%)** | | | |
| Not applicable | 9 (15.8) | 1 (5.0) | 8 (21.6) |
| No | 43 (75.4) | 19 (95.0) | 24 (64.9) |
| Yes | 5 (8.8) | 0 (0.0) | 5 (13.5) |

**Supplementary Table S1. Additional baseline histologic, staging, and prior treatment characteristics stratified by treatment intent.**

| **Characteristic** | **First-line palliative n=27** | **Second-line palliative n=10** |
| --- | --- | --- |
| **ICI regimen, n (%)** | | |
| Pembrolizumab | 16 (59.3) | 3 (30.0) |
| Nivolumab | 2 (7.4) | 3 (30.0) |
| Nivolumab plus ipilimumab | 9 (33.3) | 4 (40.0) |
| **ECOG performance status, n (%)** | | |
| ECOG 0 | 4 (14.8) | 3 (30.0) |
| ECOG 1 | 19 (70.4) | 6 (60.0) |
| ECOG 2 | 3 (11.1) | 1 (10.0) |
| ECOG 3 | 1 (3.7) | 0 (0.0) |
| **BRAF status, n (%)** | | |
| Mutated | 2 (7.4) | 5 (50.0) |
| Wild type | 23 (85.2) | 2 (20.0) |
| Not reported | 2 (7.4) | 3 (30.0) |
| **Melanoma subtype, n (%)** | | |
| Cutaneous | 7 (25.9) | 5 (50.0) |
| Acral | 11 (40.7) | 1 (10.0) |
| Mucosal | 8 (29.6) | 2 (20.0) |
| Uveal | 0 (0.0) | 1 (10.0) |
| Unknown primary | 1 (3.7) | 1 (10.0) |
| **LDH category, n (%)** | | |
| Below institutional range (<140 U/L) | 2 (7.4) | 0 (0.0) |
| Within institutional range (140–271 U/L) | 10 (37.0) | 3 (30.0) |
| Elevated (>271 U/L) | 3 (11.1) | 6 (60.0) |
| Not available | 12 (44.4) | 1 (10.0) |
| **CNS involvement, n (%)** | | |
| No | 25 (92.6) | 9 (90.0) |
| Yes | 2 (7.4) | 1 (10.0) |
| **Hepatic involvement, n (%)** | | |
| No | 24 (88.9) | 7 (70.0) |
| Yes | 3 (11.1) | 3 (30.0) |

**Supplementary Table S2. Baseline characteristics of patients treated with first-line versus second-line palliative-intent immunotherapy.**

### Supplementary Figure S1. Survival outcomes according to acral versus non-acral melanoma in the adjuvant-intent cohort. Exploratory Kaplan–Meier estimates of (A) rwDFS and (B) rwOS in patients with acral versus non-acral melanoma treated with adjuvant-intent immune checkpoint inhibitors.

**Supplementary Figure S2.** Survival outcomes according to conventional cutaneous versus acral melanoma in the palliative-intent cohort. Exploratory Kaplan–Meier estimates of (A) rwPFS and (B) rwOS in patients with conventional cutaneous versus acral melanoma treated with palliative-intent immune checkpoint inhibitors. The analysis was restricted to patients with conventional cutaneous or acral melanoma; patients with mucosal, uveal, or unknown-primary melanoma were excluded from this comparison.

**Supplementary Figure S3**. Survival outcomes according to immune-related adverse event occurrence. Exploratory Kaplan–Meier estimates according to irAE occurrence: (A) rwDFS and (B) rwOS in the adjuvant-intent cohort, and (C) rwPFS and (D) rwOS in the palliative-intent cohort. Shaded areas represent 95% confidence intervals, vertical tick marks indicate censored observations, and the numbers below each panel indicate patients at risk. Two-sided log-rank tests were used to compare survival distributions. Because irAEs occur after treatment initiation and were analyzed as a fixed exposure, these analyses are susceptible to immortal-time bias and should be interpreted as exploratory and hypothesis-generating.

| **Treatment group** | **Total** | **Evaluable** | **Missing** | **CR, n (%)** | **PR, n (%)** | **SD, n (%)** | **PD, n (%)** | **ORR, n/N (%; 95% CI)** | **DCR, n/N (%; 95% CI)** |
| --- | --- | --- | --- | --- | --- | --- | --- | --- | --- |
| Palliative cohort, all | 37 | 31 | 6 | 6 (19.4) | 6 (19.4) | 5 (16.1) | 14 (45.2) | 12/31 (38.7; 23.7-56.2) | 17/31 (54.8; 37.8-70.8) |
| First-line palliative, all | 27 | 22 | 5 | 4 (18.2) | 5 (22.7) | 3 (13.6) | 10 (45.5) | 9/22 (40.9; 23.3-61.3) | 12/22 (54.5; 34.7-73.1) |
| First-line, anti-PD-1 monotherapy | 18 | 15 | 3 | 3 (20.0) | 3 (20.0) | 3 (20.0) | 6 (40.0) | 6/15 (40.0; 19.8-64.3) | 9/15 (60.0; 35.7-80.2) |
| First-line, nivolumab plus ipilimumab | 9 | 7 | 2 | 1 (14.3) | 2 (28.6) | 0 (0.0) | 4 (57.1) | 3/7 (42.9; 15.8-75.0) | 3/7 (42.9; 15.8-75.0) |
| Second-line palliative, all | 10 | 9 | 1 | 2 (22.2) | 1 (11.1) | 2 (22.2) | 4 (44.4) | 3/9 (33.3; 12.1-64.6) | 5/9 (55.6; 26.7-81.1) |
| Second-line, anti-PD-1 monotherapy | 6 | 6 | 0 | 2 (33.3) | 0 (0.0) | 2 (33.3) | 2 (33.3) | 2/6 (33.3; 9.7-70.0) | 4/6 (66.7; 30.0-90.3) |
| Second-line, nivolumab plus ipilimumab | 4 | 3 | 1 | 0 (0.0) | 1 (33.3) | 0 (0.0) | 2 (66.7) | 1/3 (33.3; 6.1-79.2) | 1/3 (33.3; 6.1-79.2) |

**Supplementary Table S3.** Best documented radiologic response, objective response rate, and disease control rate in the palliative-intent cohort, according to treatment line and immune checkpoint inhibitor regimen.

Response was based on the best documented radiologic response in routine clinical practice and did not represent blinded central RECIST review. Percentages for complete response, partial response, stable disease, and progressive disease were calculated using evaluable patients as the denominator. ORR was defined as a complete response plus a partial response. DCR was defined as complete response plus partial response plus stable disease. Missing response assessments were excluded from ORR and DCR denominators. Confidence intervals were calculated using the Wilson method and should be interpreted cautiously in small subgroups.

**Note.** Percentages for CR, PR, SD, PD, ORR, and DCR were calculated using evaluable patients as the denominator. Confidence intervals were estimated using the Wilson method. Six patients had no valid post-treatment radiologic assessment. CR, complete response; DCR, disease control rate; ORR, objective response rate; PD, progressive disease; PR, partial response; SD, stable disease.

| **Characteristic** | **Adjuvant intent (n = 20)** | **Palliative intent (n = 37)** |
| --- | --- | --- |
| Any irAE, n (%) | 11 (55.0) | 20 (54.1) |
| **Maximum grade, n (%)** |  |  |
| Grade 1 | 4 (20.0) | 5 (13.5) |
| Grade 2 | 3 (15.0) | 9 (24.3) |
| Grade 3 | 3 (15.0) | 2 (5.4) |
| Non-classifiable or undocumented | 1 (5.0) | 4 (10.8) |
| **Organ system involved, n (%)** |  |  |
| Endocrine | 7 (35.0) | 15 (40.5) |
| Gastrointestinal | 4 (20.0) | 7 (18.9) |
| Cutaneous | 2 (10.0) | 3 (8.1) |
| Rheumatologic | 1 (5.0) | 2 (5.4) |
| Pulmonary | 1 (5.0) | 0 (0.0) |
| **Specific documented events, n (%)** |  |  |
| Primary adrenal insufficiency | 4 (20.0) | 6 (16.2) |
| Primary hypothyroidism, clinical | 2 (10.0) | 4 (10.8) |
| Subclinical hypothyroidism | 1 (5.0) | 3 (8.1) |
| Hypophysitis | 0 (0.0) | 3 (8.1) |
| Transaminase elevation without hyperbilirubinemia | 3 (15.0) | 4 (10.8) |
| Transaminase elevation with hyperbilirubinemia | 0 (0.0) | 1 (2.7) |
| Diarrhea/colitis | 1 (5.0) | 2 (5.4) |
| Thyrotoxicosis | 1 (5.0) | 0 (0.0) |
| De novo hyperglycemia | 0 (0.0) | 1 (2.7) |

**Supplementary Table S4**. Immune-related adverse events according to treatment intent, organ system, specific event, and maximum grade.

Percentages were calculated using the total number of patients in each treatment-intent cohort as the denominator. Severity was graded according to CTCAE version 5.0 when available or retrospectively inferred from clinical documentation. NC indicates events with insufficient information to assign a specific CTCAE grade. Event-specific categories were not mutually exclusive because individual patients could experience more than one irAE. No grade 4 or grade 5 irAEs were documented.

**Abbreviations:** CI, confidence interval; CTCAE, Common Terminology Criteria for Adverse Events; G, grade; irAE, immune-related adverse event; NC, not classifiable.

| **Characteristic** | **irAE present n=20** | **irAE absent n=17** |
| --- | --- | --- |
| Age, years, median (IQR) | 62.5 (51.3–75.3) | 67.0 (55.0–74.0) |
| **Sex, n (%)** |  |  |
| Female | 13 (65.0) | 9 (52.9) |
| Male | 7 (35.0) | 8 (47.1) |
| **ECOG performance status, n (%)** |  |  |
| 0 | 4 (20.0) | 3 (17.6) |
| 1 | 13 (65.0) | 12 (70.6) |
| 2 | 2 (10.0) | 2 (11.8) |
| 3 | 1 (5.0) | 0 (0.0) |
| **Melanoma subtype, n (%)** |  |  |
| Conventional cutaneous | 10 (50.0) | 2 (11.8) |
| Acral | 3 (15.0) | 9 (52.9) |
| Mucosal | 6 (30.0) | 4 (23.5) |
| Uveal | 0 (0.0) | 1 (5.9) |
| Unknown primary | 1 (5.0) | 1 (5.9) |
| **BRAF status, n (%)** |  |  |
| Mutated | 5 (25.0) | 2 (11.8) |
| Wild type | 13 (65.0) | 12 (70.6) |
| Not reported | 2 (10.0) | 3 (17.6) |
| **LDH category, n (%)** |  |  |
| Within institutional range | 7 (35.0) | 6 (35.3) |
| Elevated | 4 (20.0) | 5 (29.4) |
| Below institutional range | 1 (5.0) | 1 (5.9) |
| Not available | 8 (40.0) | 5 (29.4) |
| CNS involvement, n (%) | 2 (10.0) | 1 (5.9) |
| Hepatic involvement, n (%) | 3 (15.0) | 3 (17.6) |
| **Treatment line, n (%)** |  |  |
| First-line palliative | 14 (70.0) | 13 (76.5) |
| Second-line palliative | 6 (30.0) | 4 (23.5) |
| **ICI regimen, n (%)** |  |  |
| Pembrolizumab | 11 (55.0) | 8 (47.1) |
| Nivolumab | 3 (15.0) | 2 (11.8) |
| Nivolumab plus ipilimumab | 6 (30.0) | 7 (41.2) |

**Supplementary Table S6.** Baseline characteristics of the palliative-intent cohort according to immune-related adverse event occurrence.

Characteristics are summarized descriptively without hypothesis testing. irAE occurrence is a post-baseline exposure; this table is intended only to show potential baseline imbalance and does not establish comparability between groups.

Abbreviations: CNS, central nervous system; ECOG, Eastern Cooperative Oncology Group; ICI, immune checkpoint inhibitor; irAE, immune-related adverse event; IQR, interquartile range; LDH, lactate dehydrogenase.

| **Model** | **Outcome** | **Variable** | **Adjusted HR (95% CI)** | **p** | **Global Schoenfeld p** |
| --- | --- | --- | --- | --- | --- |
| Restricted conventional cutaneous vs acral | rwPFS | ECOG ≥2 vs 0–1 | 1.30 (0.37-4.60) | 0.687 | 0.961 |
| Restricted conventional cutaneous vs acral | rwPFS | Conventional cutaneous vs acral melanoma | 0.34 (0.12-0.92) | 0.034 | 0.961 |
| Restricted conventional cutaneous vs acral | rwOS | ECOG ≥2 vs 0–1 | 1.38 (0.37-5.24) | 0.632 | 0.550 |
| Restricted conventional cutaneous vs acral | rwOS | Conventional cutaneous vs acral melanoma | 0.32 (0.10-1.06) | 0.062 | 0.550 |
| Fixed-exposure irAE model | rwPFS | ECOG ≥2 vs 0–1 | 2.40 (0.78-7.44) | 0.129 | 0.263 |
| Fixed-exposure irAE model | rwPFS | irAE yes vs no | 0.36 (0.16-0.83) | 0.016 | 0.263 |
| Fixed-exposure irAE model | rwOS | ECOG ≥2 vs 0–1 | 4.22 (1.21-14.76) | 0.024 | 0.281 |
| Fixed-exposure irAE model | rwOS | irAE yes vs no | 0.20 (0.07-0.56) | 0.002 | 0.281 |

**Supplementary Table S5**. Exploratory multivariable Cox proportional hazards models in the palliative-intent cohort.

Note. The restricted conventional cutaneous-versus-acral models included 24 patients and adjusted for ECOG performance status. The fixed-exposure irAE models included 37 patients and adjusted for ECOG performance status. Because irAE occurrence was modeled as a fixed post-baseline exposure, these estimates are susceptible to immortal-time bias and should be interpreted as exploratory and hypothesis-generating. Global Schoenfeld tests showed no evidence of proportional hazards violation in any exploratory model.

Abbreviations: CI, confidence interval; ECOG, Eastern Cooperative Oncology Group; HR, hazard ratio; irAE, immune-related adverse event; rwOS, real-world overall survival; rwPFS, real-world progression-free survival.
